# Associations between Difficulty in Accessing Maternal and Child Health Services and Stress Responses among Mothers Raising Young Children during the COVID-19 Pandemic in Japan

**DOI:** 10.3390/nursrep14010039

**Published:** 2024-02-26

**Authors:** Noriko Toyama, Chikako Hokama, Misuzu Takahara, Yuko Toyama

**Affiliations:** 1 School of Health Sciences, Faculty of Medicine, University of the Ryukyus, Okinawa 903-0215, Japan; 2 Education and Research Institute for Death Control and Prevention, Graduate School of Medicine, Osaka University, Osaka 565-0871, Japan

**Keywords:** stress response, health service access, maternal and child health, mothers raising young children, COVID-19 pandemic

## Abstract

In Japan, maternal and child health (MCH) services were canceled or limited during the COVID-19 pandemic, potentially damaging the mental health of mothers raising young children. This study aimed to examine associations between difficulty in accessing MCH services and various stress responses among such mothers in Japan. An Internet-based questionnaire survey was conducted in November 2022 targeting mothers raising young children who had registered with a Japanese online research company (*n* = 1032). The questionnaires included items from the Public Health Research Foundation Stress Checklist (Short Form) (PHRF-SCL(SF)), sociodemographic variables, and questions about difficulty accessing MCH services due to COVID-19. Chi-squared tests and multivariate logistic regression analysis were performed. In total, 45.7% of mothers experienced difficulty in accessing MCH services due to COVID-19. Mean PHRF-SCL(SF) scores were 4.9 for anxiety, 7.3 for tiredness, 2.8 for autonomic symptoms, and 5.2 for depression. These scores indicated worse stress responses than the general population and mothers raising young children before the COVID-19 pandemic. Mothers who experienced difficulty in accessing MCH services due to COVID-19 were 1.47–1.53 times more likely to be associated with high stress responses (PHRF-SCL(SF) scores). Given the difficulty mothers faced accessing MCH services due to COVID-19 and the negative impact this had, careful follow-up and support are necessary for mothers affected by the COVID-19 pandemic.

## 1. Introduction

Maternal mental health problems have been found to influence children’s psychosocial and emotional development [1,2]. Significant associations have also been reported between maternal mental health and abusive behavior [3]. During the COVID-19 pandemic, many people reported decreased mental health; among pregnant and postpartum mothers, an increase in depressive symptoms was particularly evident [4,5]. In Canada, higher levels of anxiety and depressive symptoms were reported among pregnant women and mothers raising children aged 0 to 8 years [6]. In the UK, mental health was reportedly worse during the pandemic than before it among women and mothers with young children [7]. In China, higher levels of depressive symptoms were reported among pregnant women [8].

In Japan, the Maternal and Child Health (MCH) Law aims to support all children, from infancy to the beginning of elementary school, and their mothers/families through the provision of infant/child health checkups, home visits, childcare consultations, and childcare classes; these services are offered by municipalities [9]. A previous study conducted in Suzuka city (Japan) in 2015 reported that women who frequently used MCH services (including home visits, counseling from a public health nurse, and attending maternal classes) showed significantly lower Edinburgh Postnatal Depression Scale scores than women with lower use of MCH services [10]. However, during the COVID-19 pandemic, the Japanese government declared a national state of emergency and called for voluntary cooperation in refraining from going out and holding events to reduce the spread of infection [11]. Accordingly, MCH services were canceled or limited, leading to difficulty in accessing various support services for mothers raising young children. Studies conducted during the COVID-19 pandemic reported a lack of availability of social resources and parenting support services as well as a decrease in the number of counseling centers [12,13]. Furthermore, changes in circumstances and perceptions during the COVID-19 outbreak were reportedly significantly associated with the development of depressive and anxiety symptoms in mothers with young children [14]. Sociodemographic factors, such as age, economic status, employment status, child characteristics, and social support, have also been reported to influence maternal mental health during the COVID-19 pandemic [6,7,8]. However, no studies so far have reported an association between the difficulty in accessing MCH services and maternal mental health. It is thus important to obtain information to help identify ways to support mothers with young children affected by the COVID-19 pandemic.

To address this question, this study aimed to examine the associations between difficulty in accessing MCH services and various stress responses among mothers raising young children during the COVID-19 pandemic in Japan.

## 2. Materials and Methods

### 2.1. Participants and Procedure

An online questionnaire survey was conducted in November 2022. In Japan, the prevalence of COVID-19 varied by region [15]. Thus, in order to consider regional differences, the survey was conducted in four prefectures. Specifically, it targeted mothers raising young children (1–3 years old) living in Okinawa, Kagoshima, Fukuoka, or Tokyo who had registered with a Japanese online research company (Macromill, Inc., Minato, Tokyo). Since the number of registrants in Okinawa was the lowest among the four prefectures, the sample size for the other prefectures was based on the number of registrants there.

### 2.2. Measurements

The outcome variables in this study were mothers’ stress responses, which were measured using the Public Health Research Foundation Stress Checklist (Short Form) (PHRF-SCL(SF)) [16]. The PHRF-SCL(SF) consists of four subscales: anxiety, tiredness, autonomic symptoms, and depression. Each subscale consists of 6 items (24 items in total). Responses are rated on a 3-point Likert scale (0 = “Never”, 1 = “Sometimes”, and 2 = “Often”), giving total scores from 0 to 12 points for each subscale. In the analysis, scores were divided into two groups based on median values.

Other variables included the mothers’ age (age was categorized into 20–29, 30–39, and 40–49 years), working status (full-time, part-time, not working/maternity leave, or other), difficulty in accessing MCH services due to COVID-19 (yes/no), and residential area (the four prefectures). The questionnaire also asked a subjective question about economic status, i.e., whether respondents feel financially secure. Responses are rated on a 4-point Likert scale (1 = “I don’t feel”, 2 = “Somewhat I don’t feel”, 3 = “Somewhat I feel”, and 4 = “I feel”) and, subsequently, those who rated 1 or 2 were categorized into the low group and those who rated 3 or 4 were categorized into the high group in the analysis. Finally, mothers were asked about the difficulty of raising children; this was measured using the sub-items of the Child Care Stressor Scale created by Yoshinaga [17]. The scale consists of five items, including “crying often and being difficult to soothe”, “having tantrums”, “easily changing his/her mood”, “grumbling when left alone”, and “following his/her parents around and wanting to be held by them.” Responses are rated on a 4-point Likert scale (1 = “Almost never”, 2 = “Somewhat never”, 3 = “Somewhat often”, and 4 = “Always”), and total scores range from 5 to 20. In the analysis, this variable is categorized into two groups based on median values. As mentioned above, participants were from the four prefectures of Okinawa, Tokyo, Fukuoka, and Kagoshima. Okinawa is located in the southwest of Japan; it had the highest infection rate per population (407.0 per 1000) among all 47 prefectures in Japan [13]. Tokyo, the capital of Japan, had the second-highest rate of COVID-19 infection (324.7/1000 [15]). Fukuoka, a major city in the Kyushu–Okinawa region, had the fifth-highest COVID-19 infection rate [15]. Kagoshima is a neighboring prefecture to Okinawa; it had the 10th highest COVID-19 infection rate [15].

Regarding the utilization of and satisfaction with MCH services, the following five service items were selected: infant/child health checkups, home visits by public health nurses/midwives, child-rearing classes, childcare consultation, and ICT services (apps/social media) provided by the government. Mothers evaluated their satisfaction with MCH services on a three-point scale (1 = “satisfied”, 2 = ”neutral”, and 3 = ”dissatisfied”). Those who said they were dissatisfied with MCH services were asked why. Additionally, mothers who had difficulties accessing MCH services due to the COVID-19 pandemic were asked to provide the details of their experiences.

### 2.3. Data Analysis

The Chi-squared test was performed to examine bivariate associations between stress responses (PHRF-SCL(SF) scores) and the other factors. Multivariate logistic regression analysis was performed to calculate adjusted odds ratios (ORs) and 95% CIs using PHRF-SCL(SF) scores as the dependent variables; difficulty in accessing MCH services due to COVID-19 as the independent variable; and age, working status, economic status, difficulty in raising children, and prefecture as the adjusted variables. SPSS version 26 was used for this analysis. Statistical significance was set as *p* < 0.05.

### 2.4. Ethics

Ethical approval was obtained from the University of the Ryukyus (approval number: 22-2024-01-00-00).

## 3. Results

### 3.1. Characteristics of the Participants

**Figure 1:**
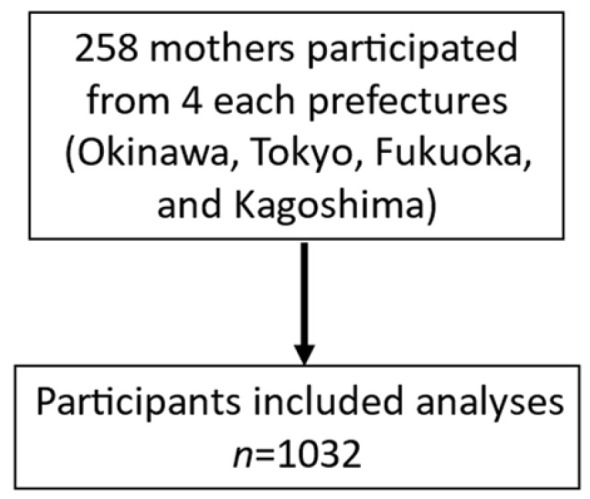
Flow chart of the study participants.

In total, 1032 mothers participated in the survey (258 from each prefecture) (Figure 1). Table 1 shows their characteristics. Regarding age, 67% were aged between 30 and 39 years. In total, 45.7% reported that they had difficulty accessing MCH services due to COVID-19.

### 3.2. Stress Responses of Mothers (Prevalence of PHRF-SCL(SF) Scores)

The Cronbach’s alpha values for the PHRF-SCL(SF) scores were 0.85 for anxiety, 0.85 for tiredness, 0.80 for autonomic symptoms, and 0.81 for depression. Mean scores (standard deviation and median) were as follows: anxiety = 4.9 (±6.0, 5), tiredness = 7.3 (±6.0, 7), autonomic symptoms = 2.8 (±1.0, 2), and depression = 5.2 (±5.0, 5).

### 3.3. Utilization and Satisfaction with and Difficulty Accessing MCH Services during the COVID-19 Pandemic

Table 2 presents the utilization of MCH services. Three-quarters of respondents utilized infant/child health checkups, while 46.5% utilized home visits by public health nurses/midwives.

Table 3 presents the satisfaction with MCH services. Fewer than 10% of mothers were satisfied with all forms of MCH services. Regarding dissatisfaction, approximately 30% of the mothers were dissatisfied with home visits, childcare classes, and childcare consultations.

Table 4 presents the main reasons for dissatisfaction with MCH services. These included COVID-19-related reasons, lack of expertise, inappropriate management, lack of explanation, and so on.

Those who had experienced difficulties in accessing MCH services due to the COVID-19 pandemic were asked to provide details of their experiences. The main answers are presented in Table 5. The most common problems were the cancellation of services and the closure of facilities. The next most common problem was restrictions on access to services, including limits on the number of people, time, and frequency.

### 3.4. Bivariate Associations between Stress Responses (PHRF-SCL(SF) Scores) and Other Variables

The results of the chi-squared test showed that all stress responses (PHRF-SCL(SF) scores) were associated with economic status, difficulty in raising children, and difficulty in accessing MCH services due to COVID-19 (Table 6).

### 3.5. Multivariate Associations of Stress Responses (PHRF-SCL(SF) Scores) with Difficulty Accessing MCH Services Due to COVID-19

The multivariate logistic regression analysis showed that mothers experiencing difficulty in accessing MCH services due to COVID-19 were 1.47–1.53 times more likely to be associated with high stress responses (PHRF-SCL(SF) scores) (Table 7).

## 4. Discussion

In this study, the mean PHRF-SCL(SF) scores were higher than those of studies conducted before the COVID-19 pandemic in the general population (3.2 for anxiety, 4.7 for tiredness, 2.2 for autonomic symptoms, and 3.7 for depression) and in mothers raising young children (3.5 for anxiety, 6.0 for tiredness, 1.4 for autonomic symptoms, and 3.3 for depression) [16,18]. Before the pandemic, only tiredness was more than 1 point higher in mothers raising young children than in the general population. In a previous study, many mothers with young children reported tiredness, including symptoms of back pain (71%) and stiff shoulders (57%) [19]. The incidence of low back pain was high among mothers with young children due to an increase in the child’s body weight. In the present study, it exceeded the percentage among mothers raising young children before the COVID-19 pandemic by more than one point. One reason for back pain and stiff shoulders during the child-rearing period could be child-rearing burdens, such as movements to support the infant’s/child’s weight. A previous study reported that mothers with physical symptoms who received support and became aware of their conditions during child-rearing consultations became mentally calmer when their physical symptoms were reduced their relationship with their children experienced a positive change [20]. Therefore, providing information and support to help prevent pain and reduce physical symptoms in mothers during childcare may be necessary. This may encompass information on stretching, muscle strengthening, and movement (such as how to hold the baby/child and how to breastfeed) during infant/child health checkups or consultations.

According to our results, 45.6% of respondents experienced difficulty in accessing MCH services due to COVID-19, and utilization and satisfaction with MCH services were lower than in previous research and the national average [21,22]. Multivariate logistic regression analysis showed that mothers experiencing difficulty in accessing MCH services due to COVID-19 were significantly associated with high stress responses (PHRF-SCL(SF) scores). These results support previous studies showing that MCH services have a positive association with maternal mental health status [10,23,24]. Mothers raising young children have many anxieties, face a significant burden due to childcare, and often have feelings of loneliness. MCH services play an important role in helping mothers mitigate the impact of these issues. Important features of these services include checking on their children’s growth and development at infant/child health checkups, consulting with professionals during childcare consultations and home visits, learning about childcare, and meeting other mothers at childcare classes. Studies in other countries have also reported experiencing barriers to accessing health services due to the COVID-19 pandemic [25]. Almost half of adults reported difficulties in accessing health services during the previous year in Poland [26]. About half of the respondents had experienced challenges in accessing maternal and child health services since the COVID-19 pandemic, and close to a third could not access services due to the lockdown in Nigeria [27]. Users of under-5 child clinics and new family planning decreased during the first 6 months of the outbreak compared to the same period the previous year in Ethiopia [28]. The most common barriers were long waiting times and temporary closure of health facilities in Poland, while fear of disease transmission, economic hardship, and transport service disruptions and restrictions were barriers to service utilization during the COVID-19 outbreak in Ethiopia [26,28]. During the COVID-19 pandemic, mothers struggled to access services when they wanted to and were unable to have social interactions with other mothers because of the cancellation or limitation of MCH services. Various restrictions were necessary during the COVID-19 pandemic to control the spread of infection. However, this study suggests that these difficult situations increased stress responses among mothers raising young children. Therefore, more careful follow-ups and support are necessary for mothers affected by the COVID-19 pandemic. In particular, people providing support services need to understand that mothers raising young children during the COVID-19 pandemic were dissatisfied with MCH services; therefore, greater attentiveness is needed.

This study has the following limitations. First, because it was cross-sectional, we were unable to identify causal relationships between the variables. Second, the mothers’ stress responses were measured via self-assessment questionnaires. Furthermore, as previous studies did not indicate a cutoff point for the stress response scale used in this study, the median was used in the analysis. This may have increased the risk of evaluation bias and potentially impacted the responses due to recall/social desirability biases. Third, we could not obtain parental couples’ and fathers’ socio-demographic characteristics due to the limited number of questions; thus, additional data collection and analysis are needed in future studies. Fourth, since the respondents only included mothers registered with the survey company, the results may not be generalizable to all mothers. Nonetheless, this study is valuable in showing the associations between stress responses and difficulty in accessing MCH services among mothers raising young children during the COVID-19 pandemic.

## 5. Conclusions

Approximately half of the mothers experienced difficulty in accessing MCH services due to COVID-19, and these mothers were significantly more likely to show anxiety, tiredness, autonomic symptoms, and depression. During the COVID-19 pandemic, mothers struggled to access services when they wanted to and found it difficult to obtain social contact with other mothers due to the cancellation or limitation of MCH services. These difficulties appeared to increase their stress responses. Therefore, more careful and targeted follow-ups and support are necessary for mothers affected by the COVID-19 pandemic.

## Figures and Tables

**Table 1:** Characteristics of the participants (*n* = 1032).

		*n*	(%)
Age (years)			
	20–29	214	(20.7)
	30–39	691	(67.0)
	≥40	127	(12.3)
Working status			
	Full-time	250	(24.2)
	Part-time	223	(21.6)
	Not working/ maternity leave	543	(52.6)
	Other	16	(1.6)
Economic status			
	High	299	(29.0)
	Low	733	(71.0)
Difficulty in raising children			
	High	426	(41.3)
	Low	606	(58.7)
Difficulty in accessing MCH services due to COVID-19			
	Yes	472	(45.7)
	No	560	(54.3)

**Table 2:** Utilization of MCH services (*n* = 1032).

Type of MCH Services	*n*	(%)
Infant/child health checkups	770	(74.6)
Home visits by public health nurses/midwives	480	(46.5)
Childcare classes	253	(24.5)
Childcare consultations	247	(23.9)
Government ICT services	92	(8.9)

**Table 3:** Satisfaction with MCH services.

	Satisfied		Neutral		Dissatisfied		Total
Type of MCH service	*n*	(%)	*n*	(%)	*n*	(%)	*n*
Infant/child health checkups	44	(5.7)	580	(75.3)	146	(19.0)	770
Home visits by public health nurses/midwives	15	(3.1)	306	(63.8)	159	(33.1)	480
Childcare classes	6	(2.4)	170	(67.2)	77	(30.4)	253
Childcare consultations	17	(6.9)	159	(64.4)	71	(28.7)	247
Government ICT services	6	(6.5)	68	(73.9)	18	(19.6)	92

**Table 4:** Main reasons for dissatisfaction with MCH services.

Type of MCH Service	Main Reasons
Infant/child health checkups	COVID-19-related (simplification of services, lack of infection control, time limitations, and requesting young children to wear masks), lack of expertise, and inappropriate management (long waiting times, lack of explanation, and consideration)
Home visits by public health nurses/midwives	Lack of expertise, lack of explanation, and feeling of being a burden
Childcare classes	Unmet expectations, impracticality, and no time to talk
Childcare consultation	Lack of resolution, unresponsiveness, inability to have discussions, and unpleasant experience
SNS/Application information from the government	Lack of expertise, insufficient information, unable to obtain necessary information, and termination of service without handover of previous records

**Table 5:** Difficulty in accessing MCH services due to COVID-19 (*n* = 472).

	*n*	(%)
Cancellation of services and closure of facilities	73	(15.5)
Restrictions on access to services (limits on the number of people, time, and frequency)	72	(15.3)
Introduction of reservation system for services	54	(11.4)
Reduction in or simplification of services	28	(5.9)
Postponement of services	16	(3.4)
Concerns about infection	64	(13.6)
Limits on going out and infection-prevention measures	58	(12.3)

**Table 6:** Associations between stress responses (PHRF-SCL(SF) scores) and other variables (*n* = 1032).

	Anxiety					Tiredness					Autonomic Symptoms					Depression				
	Low	(466)	High	(566)		Low	(425)	High	(607)		Low	(423)	High	(609)		Low	(417)	High	(615)	
	*n*	(%)	*n*	(%)	*p*	*n*	(%)	*n*	(%)	*p*	*n*	(%)	*n*	(%)	*p*	*n*	(%)	*n*	(%)	*p*
Age (years)																				
20–29	83	(17.8)	131	(23.1)	0.01	96	(22.6)	118	(19.4)	0.46	80	(18.9)	134	(22.0)	0.40	71	(17.0)	143	(23.3)	<0.01
30–39	313	(67.2)	378	(66.8)		277	(65.2)	414	(68.2)		293	(69.3)	398	(65.4)		282	(67.6)	409	(66.5)	
≥40	70	(15.0)	57	(10.1)		52	(12.2)	75	(12.4)		50	(11.8)	77	(12.6)		64	(15.3)	63	(10.2)	
Working status																				
Full-time	116	(24.9)	134	(23.7)	0.34	107	(25.2)	143	(23.6)	0.86	100	(23.6)	150	(24.6)	0.65	97	(23.3)	153	(24.9)	0.61
Part-time	96	(20.6)	127	(22.4)		87	(20.5)	136	(22.4)		85	(20.1)	138	(22.7)		85	(20.4)	138	(22.4)	
Not working/ Maternity leave	250	(53.6)	293	(51.8)		224	(52.7)	319	(52.6)		232	(54.8)	311	(51.1)		227	(54.4)	316	(51.4)	
Others	4	(0.9)	12	(2.1)		7	(1.6)	9	(1.5)		6	(1.4)	10	(1.6)		8	(1.9)	8	(1.3)	
Economic status																				
High	177	(38.0)	122	(21.6)	<0.01	163	(38.4)	136	(22.4)	<0.01	154	(36.4)	145	(23.8)	<0.01	170	(40.8)	129	(21.0)	<0.01
Low	289	(62.0)	444	(78.4)		262	(61.6)	471	(77.6)		269	(63.6)	464	(76.2)		247	(59.2)	486	(79.0)	
Difficulty in raising children																				
High	221	(47.4)	385	(68.0)	<0.01	190	(44.7)	416	(68.5)	<0.01	211	(49.9)	395	(64.9)	<0.01	182	(43.6)	424	(68.9)	<0.01
Low	245	(52.6)	181	(32.0)		235	(55.3)	191	(31.5)		212	(50.1)	214	(35.1)		235	(56.4)	191	(31.1)	
Difficulty in accessing MCH services due to COVID-19																				
Yes	181	(38.8)	291	(51.4)	<0.01	164	(38.6)	308	(50.7)	<0.01	165	(39.0)	307	(50.4)	<0.01	161	(38.6)	311	(50.6)	<0.01
No	285	(61.2)	275	(48.6)		261	(61.4)	299	(49.3)		258	(61.0)	302	(49.6)		256	(61.4)	304	(49.4)	
Prefecture																				
Okinawa	108	(23.2)	150	(26.5)	0.54	101	(23.8)	157	(25.9)	0.15	85	(20.1)	173	(28.4)	0.02	104	(24.9)	154	(25.0)	0.11
Kagoshima	115	(24.7)	143	(25.3)		110	(25.9)	148	(24.4)		119	(28.1)	139	(22.8)		102	(24.5)	156	(25.4)	
Fukuoka	119	(25.5)	139	(24.6)		95	(22.4)	163	(26.9)		110	(26.0)	148	(24.3)		92	(22.1)	166	(27.0)	
Tokyo	124	(26.6)	134	(23.7)		119	(28.0)	139	(22.9)		109	(25.8)	149	(24.5)		119	(28.5)	139	(22.6)	

**Table 7:** Multivariate analysis linking stress responses (PHRF-SCL(SF) scores) with difficulty accessing MCH services due to COVID-19 (*n* = 1032).

		Anxiety		Tiredness		Autonomic Symptoms		Depression	
		AOR	95% CI	AOR	95% CI	AOR	95% CI	AOR	95% CI
Difficulty in accessing MCH services due to COVID-19	No	1.00		1.00		1.00		1.00	
	Yes	1.53	1.18–1.98	1.47	1.13–1.91	1.47	1.14–1.91	1.50	1.14–1.96

AOR: adjusted odds ratio, 95% CI: 95% confidence interval. Dependent variable: PHRF-SCL(SF) scores (0: low score group, 1: high score group). Independent variables: Difficulty in accessing MCH services due to COVID-19 and prefecture. Adjusted variables: age, working status, economic status, difficulty in raising children, and prefecture.

## Data Availability

The data presented in this study are available from the corresponding author upon request. The data are not publicly available due to ethical restrictions.

## Public Involvement Statement

There was no public involvement in any aspect of this research.

## Guidelines and Standards Statement

This manuscript adheres to STROBE guidelines for observational studies.
